# Genetic diversity of vector-borne zoonotic pathogens in companion dogs and cats, Tianjin, China

**DOI:** 10.3389/fvets.2024.1373178

**Published:** 2024-03-14

**Authors:** Rui Jian, Jing Xue, Ze-Yun Xu, Si-Si Chen, Fang-Ni Wang, Luanying Du, Guang-Cheng Xie, Wen-Ping Guo

**Affiliations:** Department of Pathogenic Biology, College of Basic Medicine, Chengde Medical University, Chengde, Hebei, China

**Keywords:** companion cats and dogs, *Anaplasma*, *Babesia*, *Bartonella*, *Rickettsia*, pathogens

## Abstract

**Background:**

Dogs and cats are the hosts of many vector-borne human pathogens that can be transmitted to humans. Given their direct and intimate contact with humans, companion dogs and cats are considered direct sentinels of vector-borne human pathogens. However, limited information is currently available regarding canine and feline zoonotic pathogens in China. This study detected canine and feline vector-borne human pathogens to better understand the potential risk to humans.

**Methods:**

Blood samples were collected from 275 domestic companion animals (117 dogs and 158 cats) living in Tianjin city, China, and the presence of DNA from *Anaplasma*, *Babesia*, *Bartonella*, and *Rickettsia* was detected by semi-nested polymerase chain reaction (PCR). The PCR products of the expected size were sequenced, and these newly generated sequences were subjected to BLASTN, nucleotide identity, and phylogenetic analyses.

**Results:**

A total of 24 blood samples tested positive for vector-borne pathogens in companion dogs and cats in Tianjin city, China, with a relatively low positive rate of 8.7%. Specifically, seven human pathogens, including *Rickettsia raoultii*, *Candidatus Rickettsia jingxinensis*, *Rickettsia sibirica*, *Rickettsia felis*, *Babesia venatorum*, *Bartonella tribocorum*, and *Bartonella Henselae*, were identified. In addition, *Anaplasma ovis* with zoonotic potential and *Candidatus A. cinensis* were detected.

**Conclusion:**

Our results indicate substantial genetic diversity in the vector-borne human pathogens circulating in companion dogs and cats. Interventions based on “One Health” should be taken to reduce the potential risks of contracting infection from companion dogs and cats in Tianjin, China.

## Introduction

Companion dogs and cats are considered good friends of humans, and they are treated like family and experience close contact with humans, sharing their living environment. Despite the benefits of companion cats and dogs for humans, they are actually important sources of many neglected infectious diseases, such as the rabies virus responsible for the infamous rabies. In addition, pet dogs and cats are the reservoir hosts of several vector-borne pathogens, such as *Anaplasma phagocytophilum*, *Anaplasma capra*, *Ehrlichia chaffeensis*, and *Bartonella henselae* (1–3). Furthermore, these pathogens can be transmitted from companion cats and dogs to humans. Therefore, canine and feline vector-borne zoonotic diseases can be prevented and controlled under the “One Health” concept in all aspects, including epidemiology (4).

The vector-borne pathogens of major concern in companion cats and dogs that can infect humans are the genera *Anaplasma*, *Ehrlichia*, *Rickettsia*, *Borrelia*, and *Bartonella* (4, 5). The common pathogens are *A. phagocytophilum*, which causes human granulocytic anaplasmosis (HGA); *E. chaffeensi*, which causes human monocytic ehrlichiosis (HME); *Ehrlichia ewingii*, which causes human granulocytic ehrlichiosis (HGE); *Bar. henselae*, which causes cat scratch disease; and spotted fever group rickettsiae (SFGR), which causes rickettsiosis. Many vector-borne causative agents can infect companion cats and dogs through blood-sucking arthropods (including ticks, fleas, and mites). Humans can be infested by fleas or ticks obtained in the wild and brought home by pet dogs and cats, serving as bridging hosts. Therefore, dogs and cats that are in direct contact with their owners can be considered direct sentinels for human infections, as confirmed in previous studies (2, 6–12).

In China, limited studies have been performed to determine the presence of vector-borne zoonotic pathogens in companion cats and dogs. Regarding SFGR, *Rickettsia felis* (13, 14), *Rickettsia massiliae* (15), *Rickettsia conorii* (16), *Rickettsia raoultii*, and *Candidatus Rickettsia tarasevichiae* (13) have been identified in dogs. Regarding *Bartonella*, *Bar. henselae* (17–20) and *Bartonella clarridgeiae* (20) have been detected in cats. Regarding *Anaplasma*, *Anaplasma platys*, *A. phagocytophilum*, *Anaplasma ovis*, *Anaplasma bovis*, and *Anaplasma capra* have been found in dogs (3, 21–23). To date, no human-pathogenic *Babesia* species have been identified in cats or dogs. More importantly, human cases caused by *R. felis* (24), *R. raoultii* (25), *Candidatus R. tarasevichiae* (26), *Bar. henselae* (27), *A. phagocytophilum* (28), and *A. capra* (29) have been found in China.

Tianjin city is the largest port city in northern China, with a large population and numerous pet stores. A previous study in Tianjin city showed that *Ixodes persulcatus* and *Haemaphysalis longicornis* were the predominant species, while no human pathogens were identified in them using molecular methods (30). In addition, a seroepidemiological survey indicated that antibodies against *A. phagocytophilum*, *Rickettsia sibirica*, and *Ehrlichia chaffeensis* were identified in humans (31). To date, *Bartonella* and *Babesia* have not been identified in any samples. Furthermore, no study has been performed to reveal the presence of vector-borne human pathogens in companion cats or dogs. As these animals are direct sentinels for human infections, here, a comprehensive molecular survey was conducted to assess the potential risk to humans of agents belonging to the genera *Anaplasma*, *Babesia*, *Bartonella*, and *Rickettsia* in dogs and cats in Tianjin, China.

## Materials and methods

### Blood sample collection

From March to October 2021, 275 pets (i.e., 117 dogs and 158 cats) living in urban areas (Hexi District) of Tianjin city were randomly enrolled at a veterinary medical center in Tianjin city after presentation for a general inspection. These pets were selected to detect vector-borne bacteria, and all of them were clinically healthy with no clinical signs. In addition, all of the animals were born in Tianjin city and had no traveling history outside Tianjin city in the past year. A volume of 1 mm of the EDTA-anticoagulated whole blood sample was collected from each animal by venipuncture of the jugular vein with the help of veterinarians, immediately stored in a −80°C freezer, and subsequently transported on dry ice to the laboratory of the College of Basic Medicine, Chengde Medical University. This study was approved by the Scientific Ethics Committee of Chengde Medical University (number 202004). Oral consent for blood collection was obtained from all the owners of the companion dogs and animals.

### DNA extraction and pathogen detection

The frozen blood sample was thawed, and 200 μL was used for DNA extraction using the OMEGA Blood DNA Kit (OMEGA, Norcross, GA, United States) as per the manufacturer’s instructions. The DNA was eluted in 80 μL of elution buffer and stored at −20°C until further pathogen detection.

Vector-borne pathogens were identified by detecting their DNA using semi-nested PCR. The genus *Rickettsia* was identified by amplifying the partial *ompA* gene using the primer pairs Rr190k.70p/Rr190k.720n and Rr190k.70p/Rr190k.602n (32). The genus *Babesia* was detected by amplifying the partial 18S rRNA gene using the primer pairs BS1/PiroC and PiroA/PiroC (33). The primer pairs F/R1 and F/R2 targeting the *gltA* gene were used to detect *Anaplasma ovis* (34). The primer pairs Pglt-F/Pglt-R1 and Pglt-F/Pglt-R2 targeting the *gltA* gene were used to detect *Candidatus A. cinensis* (35). The primer pairs Bar-ftsz-F1/Bar-ftsz-R (Bar-ftsz-RM) and Bar-ftsz-F2/Bar-ftsz-R (Bar-ftsz-RM) targeting the *ftsz* gene were used to detect the genus *Bartonella* (36). The *gltA* gene was also amplified using the primer pairs Bar-gltA-F/Bar-gltA-R1 and Bar-gltA-F/Bar-gltA-R2, as described by Jian et al. (36). All the primer sequences used in the present study are shown in Table 1. The PCR procedures were the same as those used in previous studies (32–36). In addition, to prevent contamination, the PCR mixture preparation, template addition, and agarose gel electrophoresis were performed in a fume hood in three separate rooms, and filter tips were also used in each assay. Furthermore, ddH_2_O was used as a negative control.

**Table 1:** Primer sequences used in this study.

Pathogens	Target gene	Run	Primer	Oligonucleotide sequences (5′- 3′)	Size of products (bp)	References
*Rickettsia*	*ompA*	First	Rr190k.70p	TGGCGAATATTTCTCCAAAA (+)	650	(32)
			Rr190k.720n	TGCATTTGTATTACCTATTGT (−)		
		Second	Rr190k.70p	TGGCGAATATTTCTCCAAAA (+)	532	
			Rr190k.602n	AGTGCAGCATTCGCTCCCCCT (−)		
*Bartonella*	*gltA*	First	Bar-gltA-F	TTACYTAYGAYCCYGGBTTTA (+)	1,086	(36)
			Bar-gltA-R1	CYTCRATCATTTCTTTCCAYTG (−)		
		Second	Bar-gltA-F	TTACYTAYGAYCCYGGBTTTA (+)	1,036	
			Bar-gltA-R2	GCAAAVAGAACMGTRAACAT (−)		
	*ftsz*	First	Bar-ftsz-F1	ATGACGATTAATCTGCATCG (+)	866/581	
			Bar-ftsz-R/Bar-ftsz-RM	TCTTCRCGRATACGATTRGC (−) /TAAAGHACTTGRTCAGCCAT (−)		
		Second	Bar-ftsz-F2	ATTAATCTGCATCGGCCAGA (+)	860/575	
			Bar-ftsz-R/Bar-ftsz-RM	TCTTCRCGRATACGATTRGC (−) /TAAAGHACTTGRTCAGCCAT (−)		
*Anaplasma ovis*	*gltA*	First	F	GTGAGCTTGCCGACTTTGT (+)	620	(34)
			R1	GTTCTTGTAGACYCTGTGG (−)		
		Second	F	GTGAGCTTGCCGACTTTGT (+)	592	
			R2	ATGAGTCTCACTCCGCTCT (−)		
*Candidatus Anaplasma cinensis*	*gltA*	First	Pglt-F	ATGAWAGAAAAWGCTGTTTT (+)	671	(35)
			Pglt-R1	TCATGRTCTGCATGCATKATG (−)		
		Second	Pglt-F	ATGAWAGAAAAWGCTGTTTT (+)	661	
			Pglt-R2	CATGCATKATGAARATCGCRT (−)		
*Babesia*	18S rRNA	First	BS1	GACGGTAGGGTATTGGCCT (+)	470	(33)
			PiroC	CCAACAAAATAGAACCAAAGTCCTAC (−)		
		Second	PiroA	ATTACCCAATCCTGACACAGGG (+)	376	
			PiroC	CCAACAAAATAGAACCAAAGTCCTAC (−)		

The PCR products were examined by electrophoretic analysis on a 1.0% agarose gel. The amplicons of the expected size were purified from agarose gels using the Takara MiniBEST Agarose Gel DNA Extraction Kit Version 4.0 (Takara, Dalian, China) and sequenced bidirectionally with the PCR primers using the ABI-PRISM Dye Termination Sequencing Kit and the ABI 3730 Genetic Analyzer.

### Nucleotide sequence analysis

All the sequences recovered in this study were subjected to BLASTN against the GenBank database to determine the similarity with known sequences. The MegAlign program in Lasergene was used to calculate the nucleotide sequence identities between the sequences in this study and reference sequences (37). PhyML 3.0 was used to reconstruct the maximum likelihood (ML) tree (38). The most adequate nucleotide substitution model (GTR + *Γ* + I) used for phylogenetic analysis was estimated by MEGA 6.0.6 (39). The bootstrap analysis with 1,000 replicates was performed to evaluate the reliability of the trees. All the sequences in this study have been deposited in GenBank, and the accession numbers corresponding to each sequence are shown in Supplementary Table S1.

### Statistical analysis

A Chi-square test was used to compare the positive rates of vector-borne pathogens infection in cats and dogs using SPSS software version 24.0 (Armonk, New York, United States), and a *p* < 0.05 was considered to indicate statistical significance.

## Results

### Detection of pathogens

The BLASTN analysis of sequences obtained using PCR showed that 24 blood samples tested positive for a diverse range of vector-borne pathogens, with a total positive rate of 8.7% (24/275). In total, seven causative agents, including *R. raoultii*, *Candidatus R. jingxinensis*, *R. sibirica*, *R. felis*, *Bab. venatorum*, *Bar. Tribocorum*, and *Bar. henselae*, were identified, as were *A. ovis* with zoonotic potential and *Candidatus A. cinensis*. Of the 24 positive samples, nine were from cats, and 15 were from dogs, with positive rates of 5.7% (9/158) and 12.8% (15/117), respectively, presenting no significant difference (*p* = 0. 38).

Specifically, 13 tested positive for *Rickettsia*, with a total positive rate of 4.7%. In detail, the positive rates of *Rickettsia* infection in cats and dogs were 3.8% (6/158) and 6.0% (7/117), respectively, showing no significant difference (*p* = 0.398). Based on the BLASTN analysis, *R. raoultii*, *Candidatus R. jingxinensis*, and *R. sibirica* were identified in dogs, and *R. raoultii*, *Candidatus R. jingxinensis*, *R. sibirica*, and *R. felis* were identified in cats. In addition, *Anaplasma* was identified in one cat (0.6%, 1/158) and three dogs (2.6%, 3/117), with no significant difference in the positive rate (*p* = 0.186). The BLASTN analysis revealed *A. ovis* infection in one cat and two dogs, as well as *Candidatus A. cinensis* infection in one dog. Furthermore, the positive rate of dogs infected with *Bab. venatorum* (1.9%, 3/158) was similar to that of cats (1.7%, 2/117). Interestingly, *Bartonella* species, including *Bar. tribocorum* and *Bar. henselae*, were detected only in dogs (1.7%, 2/117). Co-infection with different pathogens was not observed in any of the animals.

### Nucleotide sequence analysis

**Figure 1:**
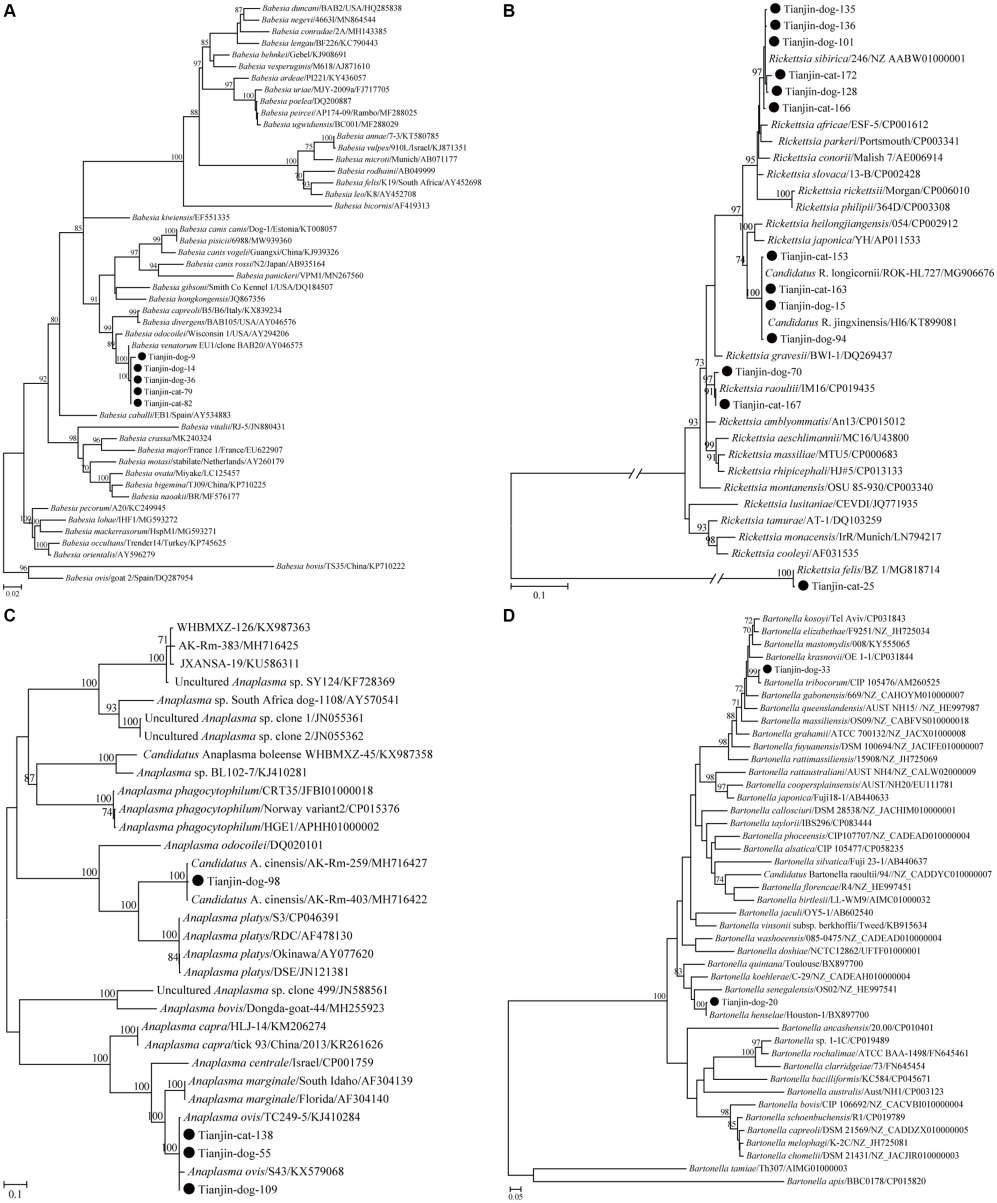
ML trees reconstructed based on the partial 18S rRNA (376 bp) gene sequences of *Babesia* **(A)**, partial *ompA* (532 bp) gene sequences of *Rickettsia* **(B)**, partial *gltA* (355 bp) gene sequences of *Anaplasma* **(C)**, and partial *ftsz* (575 bp) gene sequences of *Bartonella* **(D)**. Bootstrap values were calculated with 1,000 replicates and only >70% are shown. Sequences determined herein are marked with a black circle. The tree was mid-point rooted, and the scale bar represents the number of nucleotide substitutions per site.

Regarding *Bab. venatorum*, five partial 18S rRNA gene sequences, including two from cats and three from dogs, shared 100% nucleotide identity. Furthermore, all five of these sequences presented the highest nucleotide identity of 99.7% with the isolate Weichang-HcBv3 (MG869297), which was identified in a tick from Weichang County of China, and more than 99.3% nucleotide identity with other *Bab. venatorum* isolates in GenBank. Consistently, these five variants clustered with other *Bab. venatorum* isolates in the phylogenetic tree (Figure 1A).

Regarding the genus *Rickettsia*, all 13 partial *ompA* gene sequences were classified into four groups in the phylogenetic tree and corresponded to *R. felis*, *Candidatus R. jingxinensis*, *R. raoultii*, and *R. sibirica* (Figure 1B). The first group contained only one sequence detected in cats that shared 100% nucleotide identity with the reference sequence of *R. felis* URRWXCal2 (CP000053) and more than 97.4% nucleotide identity with others in GenBank. The second consisted of four sequences, including two from cats and another two from dogs, presented 99.6–100% nucleotide identities with each other, and shared the highest nucleotide identities of 98.3–99.8% with those of *Candidatus R. longicornii* in GenBank. The third group contained two sequences, including one from a cat and the other from a dog, presented 99.4% nucleotide identity with each other, and shared the highest nucleotide identities of 98.6–100% with those of *R. raoultii* in GenBank. The last group comprised six sequences, including two from cats and four from dogs, and exhibited 99.6 to 100% nucleotide identities with each other and 99.2 to 100% with those of *R. sibirica* in GenBank.

Regarding the genus *Anaplasma*, two species, namely, *A. ovis* and *Candidatus A. cinensis*, were identified based on the similarity of nucleotide sequences. In the phylogenetic tree, the *Candidatus Anaplasma cinensis* isolates in this study clustered with known *Candidatus A. cinensis* variants, separated from *A. platys* (Figure 1C). A partial *gltA* gene sequence from one dog herein shared 98.6–99.5% nucleotide identities with those of *Candidatus A. cinensis* in GenBank. All three partial *gltA* gene sequences of *A. ovis* in this study clustered with those of *A.ovis* in the *gltA*-based tree (Figure 1C). All of them shared 100% nucleotide identity with each other and 99.6–100% nucleotide identities with those of *A. ovis* in GenBank.

Regarding the genus *Bartonella*, two partial sequences identified from dogs but not cats clustered with those of *Bar. tribocorum* and *Bar. henselae* in the *ftsZ*-based tree (Figure 1D). The partial *ftsZ* gene sequence of *Bar. tribocorum* obtained in this study presented 99.6–100% nucleotide identities with known ones of *Bar. tribocorum* in GenBank. Regarding *Bar. henselae*, the partial *gltA* and *ftsZ* gene sequences in the present study had 99.7–100% and 99.1–99.8%, respectively, nucleotide identities with those of *Bar. henselae* in GenBank.

## Discussion

In terms of “One health,” companion animals, including dogs and cats, play a key role in the natural transmission and maintenance of some vector-borne pathogens as hosts (40). In this study, blood samples from companion dogs and cats were collected to identify vector-borne pathogens in the urban areas of Tianjin municipality in China. The results showed nine species, namely *R. raoultii*, *Candidatus R. jingxinensis*, *R. sibirica*, *R. felis*, *Bab. venatorum*, *Bar. tribocorum*, *Bar. henselae*, *A. ovis*, and *Candidatus A. cinensis*, were identified, exhibiting substantial genetic diversity of vector-borne bacteria and protozoans locally. Because these companion cats and dogs had no traveling history outside Tianjin city in the past 1 year, these pathogens identified in this study may have originated from Tianjin, China. Alternatively, these pathogens may have come from vectors that were brought back to Tianjin city by owners traveling outside Tianjin city, although this possibility is very small. Therefore, vectors, mainly including ticks, should be collected to determine their associated pathogens in Tianjin city in future studies. The total positive rate of vector-borne pathogens infection in dogs was higher than that in cats, and dogs were also more frequently infected with *Rickettsia*. However, similar positive rates of other agents were observed in dogs and cats. Furthermore, *R. raoultii*, *Candidatus R. jingxinensis*, *R. sibirica*, *R. felis*, *Bab. venatorum*, *Bar. tribocorum*, and *Bar. henselae* were pathogenic to humans. Human infections with all these pathogens have been found in China, except for *Bar. tribocorum* (41). In addition, *A. ovis* has demonstrated zoonotic potential because it has been detected in a human case (42).

Although several *Babesia* species have been identified in cats and dogs, most are not pathogenic to humans except for *Bab. microti* (43). Furthermore, rodents are considered reservoir hosts, although *Bab. microti* has been identified in cats in several previous studies (44). *Babesia* species only infecting cats and dogs were not identified in this study, while *Bab. venatorum*, which is also an emerging human pathogen, was detected in both dogs and cats. In China, *Bab. venatorum* has been identified in *Ixodes persulcatus* in Heilongjiang Province and has caused human infections in Heilongjiang and Xinjiang Provinces (41). In addition, *Bab. venatorum* was also found in *I. persulcatus* and *Haemaphysalis concinna* removed from humans in Hebei, Chian (45). In Tianjin city, *I. persulcatus* has been found (46), and whether *Bab. venatorum* infection occurs in this tick species should be determined locally. Importantly, the risk of *Bab. venatorum* to the local population should be evaluated in future studies.

In China, more than 20 *Rickettsia* species have been identified among diverse ticks (41) as hosts of ticks, dogs, and cats are naturally at risk for rickettsial infection. To date, several investigations have been performed, and at least six *Rickettsia* species, *R. felis* (13, 14), *R. massiliae*, *Candidatus R. barbariae* (15), *R. conorii* (16), *R. raoultii*, and *Candidatus R. tarasevichiae* (13) have been identified in dogs. In addition, the positive rate of *Rickettsia* infection in dogs ranged from 0.8 to 8.0%. Although reactive antibodies against *Rickettsia* have been found in cats, DNA was not identified in any of the blood samples (14). In this study, *R. raoultii*, *Candidatus R. jingxinensis*, *R. sibirica*, and *R. felis* were identified in cats, and the former three were identified in dogs in Tianjin, China. Moreover, the positive rates in this study were similar to those in previous studies (13, 14). Interestingly, this is the first report of *Candidatus R. jingxinensis* and *R. sibirica* in cats and dogs. In addition, *R. felis* was not identified in dogs in this study, although such infections have been reported previously (14). Given that the above-mentioned four *Rickettsia* species can infect humans, more attention should be paid to investigating human infections despite the relatively low positive rate of *Rickettsia* infection in companion cats and dogs in Tianjin, China. In addition, investigations should be performed to determine the prevalence of *R. raoultii*, *Candidatus R. jingxinensis*, and *R. sibirica* in ticks and of *R. felis* in cat flea (*Ctenocephalides felis*), which will be helpful for control and prevention of rickettsial infection in humans.

To date, eight validated species have been found in the genus *Anaplasma*; two have been confirmed to be pathogenic to humans and three possess zoonotic potential. Among the genus *Anaplasma*, *A. phagocytophilum*, *A. platys*, *A. bovis*, and *Candidatus A. turritanum* have been detected in cats (2). *A. phagocytophilum*, *A. platys*, *A. bovis*, *A. ovis*, and *A. capra* have been found in dogs (3, 23, 47). In this study, *Candidatus A. cinensis* was identified in dogs, and *A. ovis* was identified in both dogs and cats. To the best of our knowledge, this is the first report on *A. ovis* and *Candidatus A. cinensis* identified in cats and dogs, respectively. Given the close relationship between *Candidatus A. cinensis* and *A. platys* and the zoonotic potential of *A. platys*, the pathogenicity of *Candidatus A. cinensis* should be evaluated in the future studies.

*Bartonella* spp. are emerging vector-borne human pathogens, and a great number of mammals, including cats and dogs, are considered to be reservoir hosts (1). To date, *Bar. henselae*, *Bar. quintana*, *Bar. koehlerae*, *Bar. Bovis*, and *Bar. clarridgeiae* have been detected in both cats and dogs. In addition, *Bar. vinsonii*, *Bar. elizabethae*, *Bar. washoensis*, *Bar. Rochalimae*, and *Bar. vinsonii* have been found in dogs (48). More importantly, all these *Bartonella* species are pathogenic to humans. Therefore, these *Bartonella* species carried by cats and dogs pose a great potential threat to human health. Cats are the primary reservoir hosts for *Bar. henselae*, which is the predominant *Bartonella* species identified in cats (49). However, dogs may act as accidental hosts for *Bar. Henselae*, although it was detected occasionally in dogs (19, 50). In this study, *Bar. henselae* was detected in one dog and not in cats, with a low positive rate, suggesting a low potential risk of *Bartonella* infection in humans. However, *Bar. henselae* has been circulating in Tianjin city, and additional studies should be conducted in the future to determine its prevalence in cats and dogs in Tianjin, China. In addition to *Bar. henselae*, *Bar. tribocorum*, an emerging human pathogen hosted by rodents, was found in one dog. To the best of our knowledge, this is the first report on *Bar. tribocorum* infections in dogs. Given the direct transmission of *Bar. henselae* from cats to humans by scratch, whether *Bar. tribocorum* can be transmitted in the same way should be concerned.

One limitation of our study is that information on the gender, age, and breed of the cats and dogs was not collected; therefore, the correlations between the positive rate and gender, age, and breed cannot be determined. In this study, another limitation is that ticks, fleas, and other arthropod vectors were not collected from the companion cats and dogs. This limitation resulted in an inability to determine the transmission risks of the pathogens identified in this study. The real risks of these pathogens identified in this study to humans should be evaluated by identifying human cases in the future studies.

In conclusion, a molecular survey of vector-borne pathogens was conducted in companion cats and dogs in Tianjin, China. Our results revealed that seven human pathogens, namely *R. raoultii*, *Candidatus R. jingxinensis*, *R. sibirica*, *R. felis*, *Bab. venatorum*, *Bar. tribocorum*, and *Bar. henselae*, are circulating in Tianjin city, but the positive rate is low. In addition, *A. ovis* with zoonotic potential and *Candidatus A. cinensis* were identified. Considering the close and direct contact between companion cats and dogs and their owners, greater efforts are needed to prevent fleas or ticks from parasitizing these animals and decrease the transmission of the above-mentioned pathogens from wild animals to companion cats and dogs and further to humans.

## Data availability statement

The datasets presented in this study can be found in online repositories. The names of the repository/repositories and accession number(s) can be found in the article/Supplementary material.

## Ethics statement

The animal studies were approved by Ethics Committee of Chengde Medical University. The studies were conducted in accordance with the local legislation and institutional requirements. Written informed consent was not obtained from the owners for the participation of their animals in this study because only blood samples were collected with the help of veterinarians, and we did not conduct any other experiments on these animals. Oral permission was granted by the owners of the pet dogs and animals for blood collection.

## Author contributions

RJ: Data curation, Investigation, Methodology, Resources, Validation, Writing – original draft, Writing – review & editing. JX: Investigation, Methodology, Resources, Validation, Writing – original draft, Writing – review & editing. Z-YX: Investigation, Methodology, Resources, Writing – original draft, Writing – review & editing. S-SC: Data curation, Investigation, Writing – original draft, Writing – review & editing. F-NW: Data curation, Investigation, Writing – original draft, Writing – review & editing. LD: Data curation, Supervision, Validation, Writing – original draft, Writing – review & editing. G-CX: Data curation, Supervision, Validation, Writing – original draft, Writing – review & editing. W-PG: Conceptualization, Formal analysis, Funding acquisition, Investigation, Resources, Validation, Writing – original draft, Writing – review & editing.
