# Supplementary material for: Genetic diversity of vector-borne zoonotic pathogens in companion dogs and cats, Tianjin, China
Source: Front Vet Sci. 2024 Mar 14;11:1373178. doi: 10.3389/fvets.2024.1373178 (PMC10973169; doi:10.3389/fvets.2024.1373178)
Supplement: Supplementary file 1 [file Table_1.DOCX]

Table S1 GenBank accession numbers of sequences obtained in this study.

|  | **Gene** | **Genbank numbers** | **Bacterial strain** |
| --- | --- | --- | --- |
| 1 | 18S rRNA | PP151898 | *Babesia venatorum* isolate Tianjin-cat-79 |
| 2 | 18S rRNA | PP151899 | *Babesia venatorum* isolate Tianjin-cat-82 |
| 3 | 18S rRNA | PP151900 | *Babesia venatorum* isolate Tianjin-dog-9 |
| 4 | 18S rRNA | PP151901 | *Babesia venatorum* isolate Tianjin-dog-14 |
| 5 | 18S rRNA | PP151902 | *Babesia venatorum* isolate Tianjin-dog-36 |
| 6 | *ftsz* | PP158578 | *Bartonella tribocorum* isolate Tianjin-dog-33 |
| 7 | *ftsz* | PP158579 | *Bartonella henselae* isolate Tianjin-dog-20 |
| 8 | *gltA* | PP158580 | *Anaplasma ovis* isolate Tianjin-dog-55 |
| 9 | *gltA* | PP158581 | *Anaplasma ovis* isolate Tianjin-dog-109 |
| 10 | *gltA* | PP158582 | *Anaplasma ovis* isolate Tianjin-cat-138 |
| 11 | *gltA* | PP158583 | *Candidatus* Anaplasma cinensis isolate Tianjin-dog-98 |
| 12 | *ompA* | PP158584 | *Rickettsia felis* isolate Tianjin-cat-25 |
| 13 | *ompA* | PP158585 | *Candidatus* Rickettsia jingxinensis isolate Tianjin-cat-153 |
| 14 | *ompA* | PP158586 | *Candidatus* Rickettsia jingxinensis isolate Tianjin-cat-163 |
| 15 | *ompA* | PP158587 | *Rickettsia sibirica* isolate Tianjin-cat-166 |
| 16 | *ompA* | PP158588 | *Rickettsia sibirica* isolate Tianjin-cat-172 |
| 17 | *ompA* | PP158589 | *Candidatus* Rickettsia jingxinensis isolate Tianjin-dog-15 |
| 18 | *ompA* | PP158590 | *Candidatus* Rickettsia jingxinensis isolate Tianjin-dog-94 |
| 19 | *ompA* | PP158591 | *Rickettsia sibirica* isolate Tianjin-dog-101 |
| 20 | *ompA* | PP158592 | *Rickettsia sibirica* isolate Tianjin-dog-128 |
| 21 | *ompA* | PP158593 | *Rickettsia sibirica* isolate Tianjin-dog-135 |
| 22 | *ompA* | PP158594 | *Rickettsia sibirica* isolate Tianjin-dog-136 |
| 23 | *ompA* | PP158595 | *Rickettsia raoultii* isolate Tianjin-cat-167 |
| 24 | *ompA* | PP158596 | *Rickettsia raoultii* isolate Tianjin-dog-70 |
